# Efficacy and Safety of Meropenem in Pregnant Women with Upper Urinary Tract Infections: A Retrospective Cohort Study in Romania

**DOI:** 10.3390/antibiotics15060610

**Published:** 2026-06-16

**Authors:** Gabriel-Ioan Anton, Rodica Radu, Emil Ceban, Carina Alexandra Bandac, Vasile Lucian Boiculese, Demetra Socolov, Adriana Grigoras, Radu-Stefan Miftode, Amalia Stefana Țimpău, Manuel Florin Rosu, Ionela-Larisa Miftode, Viorel Dragoș Radu

**Affiliations:** 1 Faculty of Medicine, Grigore T. Popa University of Medicine and Pharmacy Iasi, 700115 Iasi, Romania; anton.gabriel-ioan@d.umfiasi.ro (G.-I.A.); rodica.radu@umfiasi.ro (R.R.); carina_bandac@email.umfiasi.ro (C.A.B.); vasile.boiculese@umfiasi.ro (V.L.B.); demetra.socolov@umfiasi.ro (D.S.); a_grigoras6600@yahoo.com (A.G.); radu-stefan.miftode@umfiasi.ro (R.-S.M.); amalia.timpau@umfiasi.ro (A.S.Ț.); florin.rosu@umfiasi.ro (M.F.R.); viorel.radu@umfiasi.ro (V.D.R.); 2 Department of Mother and Child Care, Faculty of Medicine, University of Medicine and Pharmacy “Grigore T. Popa”, 700115 Iasi, Romania; 3 Department of Internal Medicine, Faculty of Medicine, University of Medicine and Pharmacy “Grigore T. Popa”, 700115 Iasi, Romania; 4 Department of Urology and Surgical Nephrology, University of Medicine and Pharmacy “Nicolae Testemitanu”, 165 Stefan cel Mare si Sfant Bd., MD-2004 Chisinau, Moldova; emil.ceban@usmf.md; 5 “C.I. Parhon” University Hospital, 700503 Iasi, Romania; 6 Department of Preventive and Interdisciplinarity, Medical Informatics and Biostatistics, Faculty of Medicine, University of Medicine and Pharmacy “Grigore T. Popa”, 700115 Iasi, Romania; 7 Department of Morphofunctional Sciences I—Anatomy, University of Medicine and Pharmacy “Gr. T. Popa”, 700115 Iasi, Romania; 8 “St. Spiridon” Emergency Hospital, 700115 Iasi, Romania; 9 “St. Parascheva” Clinical Hospital of Infectious Diseases, 700116 Iasi, Romania

**Keywords:** meropenem, pregnancy, upper urinary tract infection, pyelonephritis, urosepsis, septic shock, ceftriaxone, antibiotic safety, maternal outcomes, neonatal outcomes

## Abstract

**Introduction**: Upper urinary tract infections (UUTIs) are among the most common serious infections during pregnancy and may be associated with maternal and fetal complications. The increasing prevalence of multidrug-resistant pathogens has led to the use of broader-spectrum antibiotics, including meropenem. However, data regarding the safety and efficacy of meropenem in pregnant women remain limited. The aim of this study was to evaluate the indications, efficacy, and safety of meropenem treatment in pregnant women with UUTIs and its impact on maternal and fetal outcomes. **Methods**: We conducted a retrospective study over a 12-year period including pregnant women hospitalized with UUTIs who received meropenem. The control group consisted of pregnant women with UUTIs who were treated with ceftriaxone during the same period. **Results**: Pregnant women treated with meropenem were more frequently diagnosed in the third trimester of pregnancy (*p* = 0.01) and were more often multiparous (*p* = 0.006). Sepsis and septic shock occurred significantly more frequently in the study group (*p* < 0.01), and multivariate analysis identified them as the main indications for meropenem administration (OR 10.54, 95% CI 3.30–33.70 for sepsis; OR 3.28, 95% CI 1.01–10.62 for septic shock). Patients in the study group had a higher rate of transfer to the obstetrics clinic (*p* = 0.032), a longer duration of antibiotic therapy (*p* = 0.031), and a longer hospital stay (*p* < 0.01). No maternal deaths were reported in either group. The rate of adverse pregnancy outcomes was similar between the two groups, except for the Apgar score, which was lower in the meropenem group (*p* = 0.007). *Escherichia coli* and *Klebsiella pneumoniae* were the most frequently isolated pathogens in both groups. **Conclusions**: Meropenem therapy in pregnant women with UUTIs was mainly indicated in cases of sepsis and septic shock and was associated with favorable maternal clinical evolution, even in patients with severe infections. The rate of adverse pregnancy outcomes was similar between the two groups, although a lower Apgar score was observed in the meropenem group; the severity of illness in the meropenem group should be considered when interpreting the lower Apgar scores. Further prospective multicenter studies are needed to better evaluate the safety and clinical effectiveness of meropenem during pregnancy.

## 1. Introduction

Infectious diseases remain an important cause of maternal morbidity during pregnancy, and respiratory, genital, and urinary tract infections (UTIs) are among the most common conditions requiring antimicrobial therapy [1,2]. UTIs are particularly relevant in pregnant women, as physiological and anatomical changes in the urinary tract during pregnancy increase susceptibility to bacterial colonization and infection. If left untreated, UTIs may progress to more severe forms such as pyelonephritis, urosepsis, and septic shock and may be associated with adverse maternal and fetal outcomes [3].

Antibiotics represent the most frequently prescribed class of medications during pregnancy, with penicillins and cephalosporins being among the most commonly used agents because of their broad antibacterial spectrum and well-established safety profile [1,4]. Consequently, the appropriate selection and judicious use of antibiotics represent a key component in the management of infections during pregnancy. However, the increasing prevalence of multidrug-resistant (MDR) uropathogens has complicated the treatment of severe UTIs, particularly in patients with obstructive pyelonephritis, urosepsis, or septic shock [5,6]. Therefore, broad-spectrum antibiotics, including carbapenems such as meropenem, are increasingly used in selected severe infections despite the limited clinical data regarding their safety during pregnancy.

Exposure to antibiotics during pregnancy may be associated with potential adverse effects, especially when administered during the first trimester, when major congenital malformations may occur. In later stages of pregnancy, antibiotic exposure has been associated with growth disturbances, minor congenital anomalies, or functional developmental abnormalities [7]. In recent years, increasing antimicrobial resistance among pathogens causing UTIs, including those affecting pregnant women, has become a growing concern [5,6]. Therefore, careful antibiotic stewardship during pregnancy is essential in order to limit the development of antimicrobial resistance while minimizing potential adverse effects on both the mother and the fetus [8].

MDR uropathogens, particularly bacteria including *Escherichia coli*, *Klebsiella pneumoniae*, *Pseudomonas* spp., *Proteus* spp., *Serratia* spp., and *Staphylococcus aureus*, pose escalating challenges in pregnant populations and critically ill patients [9,10]. Alternative antimicrobial strategies show promise against resistant pathogenic bacteria [9,11]. However, carbapenem antibiotics remain essential for severe urosepsis when standard therapy fails, despite emerging resistance patterns [12].

Meropenem is a broad-spectrum carbapenem antibiotic that is considered a reserve therapy for severe infections, including UTIs caused by multidrug-resistant pathogens, urosepsis, and septic shock [13,14,15,16]. It is a hydrophilic molecule with bactericidal activity achieved through inhibition of bacterial cell wall synthesis [16,17]. Meropenem penetrates most tissues and body fluids [18], crosses the placental barrier, and is present in small amounts in breast milk [19,20]. In patients with normal renal function, meropenem has a half-life of approximately one hour and is eliminated primarily through renal excretion [21], with up to 70% of the drug being excreted unchanged in the urine [22,23]. Physiological changes during pregnancy, including increased renal blood flow, glomerular filtration rate, and alterations in volume of distribution, may influence the pharmacokinetics of renally eliminated antibiotics. While carbapenem pharmacokinetics during pregnancy have been investigated for imipenem [24], comparable data for meropenem are currently unavailable, representing an important gap in the literature. Among carbapenems, meropenem is often preferred over imipenem–cilastatin due to its more favorable safety profile and lower risk of adverse effects [25].

Currently, data regarding the safety of meropenem administration during pregnancy remain limited in the scientific literature [26]. According to the European Respiratory Society, meropenem is considered “possibly safe” during the first trimester and “probably safe” during the second and third trimesters of pregnancy [27]. In the FDA classification of drugs used during pregnancy, meropenem is included in pregnancy category B [28,29]. According to this classification, meropenem should be administered during pregnancy only when clearly necessary and when the potential benefits outweigh the possible risks to the fetus. This recommendation reflects the limited and inconclusive data available regarding the safety of meropenem use in pregnant women.

To date, meropenem has been studied in only a small number of pregnant patients. The largest study, published in 2001, included 13 pregnant women with UTIs and reported no significant drug-related adverse effects [30]. Most other reports consist of isolated clinical case reports or small case series [31,32,33]. Experimental animal studies evaluating fetal exposure have not demonstrated an increased risk of fetal toxicity [28]. However, comprehensive clinical studies evaluating the efficacy, safety, and maternal and fetal outcomes associated with meropenem administration during pregnancy are still lacking. Current clinical guidelines do not recommend termination of pregnancy after exposure to meropenem but advise careful fetal monitoring during pregnancy [8].

Given the limited data available in the literature, we conducted a retrospective study to evaluate the indications, efficacy, and safety of meropenem administration in pregnant patients with upper urinary tract infections (UUTIs), as well as its impact on maternal and fetal outcomes. In addition, we aimed to identify the clinical characteristics of pregnant patients in whom meropenem therapy was indicated and to analyze the bacterial spectrum responsible for UUTIs requiring treatment with meropenem.

## 2. Results

After applying the selection criteria, 40 pregnant patients who received meropenem and 127 patients treated with ceftriaxone were included in the study. Among them, 33 patients in the meropenem group and 89 in the ceftriaxone group delivered at Cuza Vodă Hospital. The baseline characteristics of the patients in the two groups are presented in Table 1.

No statistically significant differences were observed between the two groups with regard to age, side of infection (right vs. left), place of origin (urban vs. rural), or comorbidities. In both groups, UUTIs were predominantly right-sided, and most patients were older than 25 years. Although the proportion of patients from urban areas was higher in the meropenem group than in the ceftriaxone group, this difference was not statistically significant.

However, patients in the meropenem group were more frequently diagnosed during the third trimester of pregnancy (*p* = 0.01), whereas those in the ceftriaxone group were more often diagnosed during the second trimester (*p* = 0.042). Nulliparous patients were more common in the ceftriaxone group (*p* = 0.006).

The clinical conditions leading to antibiotic therapy in the two groups are presented in Table 2.

No statistically significant differences were observed between the two groups regarding the underlying diagnoses for which antibiotic therapy was initiated. Infected gestational hydronephrosis (*p* = 0.44), infected obstructive reno-ureteral lithiasis (*p* = 0.82), and reflux pyelonephritis (*p* = 0.062) occurred in similar proportions in both groups.

However, patients in the meropenem group more frequently presented at admission with sepsis (*p* < 0.01) and septic shock (*p* < 0.01). Multivariate analysis identified sepsis and septic shock as the main factors associated with the indication for meropenem therapy (Table 3).

Table 4 summarizes the clinical and laboratory characteristics of the patients in the two groups.

Patients in the meropenem group presented with higher leukocyte counts at admission (*p* < 0.01), higher CRP levels (*p* < 0.01), and higher serum creatinine values (*p* = 0.036). Fever above 38 °C was also more frequent in this group (*p* < 0.01), as was the rate of transfer to the obstetrics clinic (*p* = 0.032). In addition, patients treated with meropenem had a longer duration of hospitalization (*p* < 0.01) and a longer duration of injectable antibiotic therapy compared with those treated with ceftriaxone (*p* = 0.031).

No statistically significant differences were observed between the two groups regarding the presence of infections caused by MDR pathogens (*p* = 0.16). Similarly, the rate of readmissions associated with a positive urine culture did not differ significantly between the two groups (*p* = 0.47). Urological surgical interventions associated with antibiotic therapy occurred at similar rates in both groups (*p* = 0.99).

No adverse effects related to meropenem were reported, such as redness at the injection site or nausea. Although thrombocytopenia has been described in patients with renal impairment, no such events were observed in the meropenem group despite the higher serum creatinine levels. In the ceftriaxone group, three cases of redness at the venous puncture site were reported. No allergic reactions were observed in any of the pregnant women included in the study.

These findings confirm that patients in the meropenem group had more severe clinical presentations at baseline than those treated with ceftriaxone.

Urine cultures identified the causative pathogen in 32 patients in the meropenem group (80%) and in 86 patients in the ceftriaxone group (67%). Although culture positivity was numerically higher in the meropenem group, the difference was not statistically significant (*p* = 0.17), suggesting comparable microbiological sampling between groups. The microorganisms responsible for UUTIs in the two groups are presented in Table 5.

*Escherichia coli* was the most frequently identified pathogen in both groups, with no statistically significant difference between them (*p* = 0.35). *Klebsiella pneumoniae* was detected significantly more frequently in the meropenem group than in the ceftriaxone group (*p* = 0.031). The other pathogens were identified in very small proportions, more commonly in the ceftriaxone group, but without statistically significant differences. Detailed antimicrobial resistance patterns are presented in Supplementary Table S1.

Pregnancy outcomes among the patients who delivered at “Cuza Vodă” Hospital are presented in Table 6.

No statistically significant differences were observed between the two groups regarding the analyzed pregnancy outcomes, except for the Apgar score, which was lower in the meropenem group (*p* = 0.007). Birth weight (*p* = 0.414), rate of cesarean delivery (*p* = 1), rate of preterm birth (*p* = 0.428), and gestational age at delivery (*p* = 0.325) were similar in both groups.

Neonatal intensive care unit (NICU) admission data were available for 33/40 neonates in the meropenem group and 89/127 neonates in the ceftriaxone group. In the complete-case analysis, NICU admission was more frequent in the meropenem group than in the ceftriaxone group (23/33, 69.7% vs. 46/89, 51.7%); however, the difference did not reach statistical significance (OR 2.15, 95% CI 0.92–5.03, *p* = 0.10). Given the presence of missing neonatal outcome data, these findings should be interpreted with caution.

No maternal deaths were reported in either group. Similarly, no statistically significant difference in fetal mortality was observed between the two groups (*p* = 0.17).

## 3. Discussion

Antibiotics represent one of the most important therapeutic tools in modern medicine and are among the most frequently prescribed medications during pregnancy [34]. In pregnant women, bacterial infections may lead not only to maternal complications but also to significant adverse fetal and neonatal outcomes, making prompt and effective antimicrobial therapy essential [35]. In recent years, the global increase in antimicrobial resistance has complicated the management of infectious diseases, including UTIs [36]. This phenomenon has been further amplified during and after the COVID-19 pandemic, when widespread antibiotic use contributed to concerns regarding the acceleration of MDR pathogens [37,38,39,40]. In this context, the use of broad-spectrum antibiotics, including carbapenems, has become increasingly necessary in the treatment of severe infections, even in vulnerable populations such as pregnant women.

This study evaluated the indications, efficacy, and safety of meropenem therapy in pregnant patients with UUTIs. Our results suggest that meropenem was well tolerated and not associated with an increased rate of major adverse maternal or fetal outcomes, although it was more frequently used in patients with severe infections such as urosepsis and septic shock. Favorable maternal outcomes were observed in the meropenem group, despite the higher baseline severity of illness. Importantly, no patients discontinued treatment because of adverse effects. These findings indicate that meropenem may represent a valuable therapeutic option in pregnant patients with severe UUTIs when broad-spectrum antibiotic coverage is required; however, given the non-randomized design of the study, causal inferences should be made with caution. Our results may be particularly relevant for obstetricians, urologists, and infectious disease specialists involved in the management of complicated UTIs during pregnancy.

In both groups, most patients were aged over 25 years, and the infection was predominantly right-sided. This observation is consistent with the increased frequency of gestational hydronephrosis, which is known to predispose pregnant women to UUTIs. Although a higher proportion of patients in the meropenem group originated from urban areas and more frequently presented with anemia, these differences were not statistically significant, suggesting that the choice of meropenem versus ceftriaxone was not influenced by these variables.

However, several differences were observed between the two groups. In the meropenem group, infections were more frequently diagnosed during the third trimester of pregnancy, and patients were more often multiparous. This temporal distribution of urosepsis is consistent with physiological changes in late pregnancy. In addition, previous urinary infections or prior antibiotic exposure in multiparous patients may have contributed to the clinical decision to initiate broader-spectrum antibiotic therapy, including meropenem.

Previous studies have shown that pregnant women with complicated UTIs or urosepsis often present with risk factors such as urinary tract obstruction, previous urinary infections, or urological interventions, which increase the severity of infection and the need for broad-spectrum antibiotic therapy [40,41]. Furthermore, several studies have reported that severe UTIs and urosepsis during pregnancy occur more frequently in the second and third trimesters, when physiological urinary tract changes such as ureteral dilation and urinary stasis are more pronounced [6,40]. These observations are consistent with the higher frequency of urosepsis observed in the meropenem group in our study.

In both groups, four main clinical conditions were identified for which antibiotic therapy was initiated: infected gestational hydronephrosis, infected hydronephrosis associated with obstructive reno-ureteral lithiasis, reflux pyelonephritis, and acute non-obstructive pyelonephritis. However, in the meropenem group these conditions were more frequently associated with sepsis and septic shock, suggesting that the primary indication for meropenem administration in pregnant women with UUTIs was the severe progression of the infection. In clinical practice, meropenem was therefore more often initiated at an advanced stage of disease, particularly in cases complicated by sepsis or septic shock.

Biofilm-forming uropathogens complicate catheter-associated UTIs in pregnant women requiring urological interventions such as JJ stent placement or percutaneous nephrostomy [9,10]. Coating medical devices with antimicrobials reduces biofilm formation, though multidrug-resistant pathogenic bacterial isolates persist despite intervention [42]. Meropenem remains effective against biofilm-associated urosepsis when combined with obstruction relief.

Another important observation is that, in most patients in the meropenem group, antibiotic therapy was associated with urological interventions, such as JJ stent insertion, percutaneous nephrostomy, or replacement of an existing JJ stent. JJ stent insertion was mainly performed in patients with obstructive pyelonephritis secondary to gestational hydronephrosis or obstructive reno-ureteral lithiasis, in order to relieve urinary obstruction and achieve source control of infection. In these patients, urinary drainage represented an essential component of treatment in addition to antibiotic therapy.

The indication for meropenem in patients carrying JJ stents may be related to the increased risk of infections caused by MDR pathogens, especially considering the rising incidence of MDR infections in our region [43,44], which has been associated with prolonged JJ stent placement [45,46]. Although increasing rates of UTIs caused by carbapenem-resistant pathogens have been reported in the literature [47], none of the urine cultures in our study showed resistance to meropenem. Similar findings have been reported in other studies [40,48,49], although carbapenem-resistant UTIs in pregnant women have been described in some reports [50,51].

Among the 12 patients who developed septic shock, 6 experienced shock during JJ stent insertion. Although causality cannot be established in this retrospective study, the temporal association suggests that urinary tract instrumentation may have contributed to clinical deterioration in these patients. Several studies have also reported urosepsis and septic shock as major indications for meropenem therapy, as these severe infections are frequently caused by *Escherichia coli* or other Enterobacterales with MDR profiles [52,53,54].

The more pronounced inflammatory profile observed in the meropenem group, characterized by higher leukocyte counts, CRP levels, fever, and serum creatinine values, supports the interpretation that meropenem was preferentially administered to patients with more severe systemic infection and greater renal involvement. The higher creatinine levels may reflect renal impairment associated with sepsis, septic shock, or obstructive pyelonephritis. In routine clinical practice, elevated inflammatory markers, particularly CRP levels, were among the factors considered by treating physicians when assessing disease severity and the risk of clinical deterioration, and therefore may have contributed to the decision to initiate meropenem therapy. These findings further support the presence of confounding by indication in our cohort, as patients receiving meropenem generally presented with more severe clinical conditions compared with those treated with ceftriaxone. Importantly, despite this higher inflammatory burden and greater disease severity, maternal and fetal outcomes remained favorable among patients treated with meropenem.

This difference in disease severity may also explain the longer duration of antibiotic therapy and prolonged hospitalization observed in the meropenem group. The higher rate of transfer to the obstetrics clinic, most often due to suspected fetal distress, may be related to the increased frequency of septic shock in this group (30%), a condition known to adversely affect fetal status [41]. We do not consider that this higher transfer rate was directly related to meropenem administration; rather, meropenem may have been associated with mitigation of the negative consequences of severe infection, although not sufficiently to reduce the transfer rate to the level observed in the ceftriaxone group, where septic shock was much less frequent.

MDR pathogens were identified more frequently in the meropenem group than in the ceftriaxone group (22.5% vs. 13.38%), although the difference did not reach statistical significance (*p* = 0.16). While this trend suggests that clinical suspicion of multidrug resistance may have contributed to treatment selection in some cases, the decision to initiate meropenem was also strongly influenced by the overall severity of infection, including sepsis, septic shock, and obstructive pyelonephritis. Furthermore, ceftriaxone therapy was continued in selected patients with MDR pathogens when antimicrobial susceptibility testing demonstrated ceftriaxone susceptibility and favorable clinical evolution was observed. Therefore, the present study did not focus exclusively on MDR infections, but rather on the clinical indications, use patterns, and outcomes associated with meropenem therapy in severe UUTIs during pregnancy.

One difficulty in assessing the efficacy of the two antibiotic regimens was the fact that, in most pregnant patients, antibiotic therapy was associated with urological interventions such as JJ stent insertion, replacement of existing JJ stents, or percutaneous nephrostomy. These procedures contributed to clinical improvement in most cases by relieving urinary obstruction. However, in some situations they were followed by clinical deterioration and the development of septic shock, occurring either intraoperatively or shortly after the intervention. In such cases, ceftriaxone therapy was replaced with meropenem, suggesting that septic shock associated with urological procedures represented an important indication for the initiation of meropenem treatment. Moreover, early escalation to meropenem in clinically deteriorating patients may have contributed to limiting the progression of severe maternal infection and improving maternal–fetal outcomes.

These findings suggest that ceftriaxone did not always prevent the occurrence of sepsis or septic shock associated with severe obstructive infections and urological procedures, whereas meropenem was associated with favorable clinical evolution in these severe complications. It is also important to note that the majority of patients in both groups presented with obstructive pyelonephritis. This observation supports the hypothesis that, in the absence of urinary obstruction, pyelonephritis during pregnancy may have a more favorable clinical course, with a lower risk of progression to sepsis or septic shock and consequently a reduced need for treatment with broad-spectrum antibiotics such as meropenem.

Regarding clinical efficacy, no maternal deaths were observed in either group, suggesting a high effectiveness of meropenem even in patients presenting with sepsis or septic shock. However, the longer duration of injectable antibiotic therapy and hospitalization observed in the meropenem group should be interpreted primarily as markers of greater baseline disease severity rather than reduced treatment effectiveness. Similarly, the higher rate of transfer to the obstetrics clinic most likely reflected the increased frequency of sepsis and septic shock in these patients, conditions known to adversely influence maternal and fetal status despite appropriate anti-infectious management.

*Escherichia coli* and *Klebsiella pneumoniae* were responsible for approximately 90% of UUTIs in both groups, consistent with findings reported in other local studies [41]. Most of these pathogens were susceptible to the majority of the antibiotics tested, which may partially explain the generally favorable clinical evolution observed in our cohort, as such strains are typically associated with lower virulence and better response to antibiotic therapy. Importantly, even in infections caused by MDR pathogens, no unfavorable maternal outcomes were recorded. Furthermore, all MDR isolates in our study showed 100% susceptibility to meropenem on antimicrobial susceptibility testing, despite reports in the literature describing the emergence of carbapenem-resistant pathogens causing UTIs in pregnant women [55].

Given the higher rate of transfer to the obstetrics clinic observed in the meropenem group, we expected to identify significant differences between the two groups regarding delivery parameters, particularly those related to neonatal outcomes. Although the meropenem group showed proportionally lower Apgar scores and higher rates of preterm birth, stillbirth and NICU admission, these differences did not reach statistical significance. It is possible that small differences exist, but the relatively limited sample size of our study may have prevented them from reaching statistical significance. Sepsis and septic shock are well recognized as major risk factors for adverse pregnancy outcomes [41].

Nevertheless, these findings should be interpreted with caution. Although the meropenem group included patients with a higher frequency of severe infections, the overall rates of major adverse pregnancy outcomes were comparable between the two groups. This observation may suggest that appropriate management of severe maternal infection, including the use of broad-spectrum antibiotics when indicated, can help achieve favorable maternal and fetal outcomes. However, because patients receiving meropenem presented with significantly greater baseline disease severity, including higher rates of sepsis and septic shock, direct comparisons between the two treatment groups are limited by confounding by indication. Therefore, our findings should not be interpreted as demonstrating superiority of meropenem over ceftriaxone, but rather as suggesting favorable clinical evolution associated with meropenem therapy despite greater baseline disease severity. In addition, treatment with meropenem did not appear to influence the mode of delivery, as the rate of cesarean section was similar in both groups. Importantly, the observed differences in neonatal outcomes are likely multifactorial and may primarily reflect the higher incidence of sepsis and septic shock in the meropenem group rather than a direct effect of the antibiotic itself.

One possible explanation for the apparently lower effectiveness of meropenem in improving neonatal parameters compared with maternal outcomes may be related to the pharmacokinetic properties of the drug. Meropenem crosses the placental barrier only partially, which may result in lower fetal concentrations than maternal levels and consequently a reduced therapeutic effect on the fetus [56]. Although clinical studies have shown that meropenem is well tolerated in neonates and can be effectively used in the treatment of severe infections, its trans-placental transfer remains limited, which may partly explain the discrepancy between maternal and neonatal outcomes observed in our study [57,58,59].

No adverse effects related to meropenem administration were observed in our study, and no patients required discontinuation of treatment for this reason. Meropenem was well tolerated, and no adverse events such as nausea, infusion-site reactions, or hematological abnormalities previously reported in the literature were observed [60,61]. Moreover, compared with alternative regimens used for severe multidrug-resistant infections, particularly colistin-based therapies, meropenem-containing regimens have been associated with a more favorable safety profile, especially with respect to renal toxicity, further supporting its use in critically ill patients [62].

This favorable safety profile may also be related to the method of administration. In our cohort, meropenem was administered as a slow intravenous infusion over 30 min rather than as a rapid injection. Furthermore, the standard dose of 1 g every 8 h was used without dose escalation, as higher doses are not known to increase antibiotic efficacy but may increase the risk of adverse effects. In other studies, the duration of meropenem therapy in pregnant patients was shorter, approximately three days [54], and no significant adverse effects were reported.

To our knowledge, this study represents one of the first analyses in the literature evaluating the indications for meropenem therapy in pregnant patients with UUTIs, as well as its efficacy and safety during pregnancy and its potential impact on maternal, fetal, and delivery outcomes.

However, several limitations should be acknowledged. First, the relatively small number of cases per year required a retrospective analysis over a 12-year period. During this time, changes in the spectrum of bacterial susceptibility may have occurred, as well as variations in clinical practice that were not fully captured in our analysis. Because of the relatively low annual number of severe UUTI cases requiring meropenem therapy in pregnant women, conducting a prospective randomized controlled trial was not feasible in our setting.

The retrospective design introduces potential confounding by indication, as patients receiving meropenem generally presented with more severe infections, limiting the ability to directly compare treatment effects between groups. Moreover, antibiotic selection was not standardized but was based on the treating physicians’ clinical judgment, taking into account disease severity, perceived risk of clinical deterioration, microbiological findings when available, and individual patient characteristics. Consequently, some patients who received meropenem may also have been considered candidates for ceftriaxone, reflecting the overlap in treatment indications commonly encountered in real-world clinical practice. Therefore, our study was not designed to demonstrate superiority between meropenem and ceftriaxone, but rather to evaluate the real-world indications, clinical evolution, and maternal–fetal outcomes associated with meropenem use in severe UUTIs during pregnancy. Nevertheless, all urine cultures included in the study demonstrated susceptibility to the antibiotics administered. In addition, the relatively limited sample size may have reduced the ability to detect small differences between the two groups.

Another limitation is that not all patients included in the study could undergo follow-up until delivery, as only a proportion of them delivered at “Cuza Vodă” Hospital. However, follow-up data were available for more than 70% of patients in both groups. Although the proportion of patients with available follow-up was slightly higher in the meropenem group, this difference was not statistically significant. Nevertheless, incomplete follow-up may have introduced a degree of selection bias and could have affected the assessment of some pregnancy outcomes. In addition, although total Apgar scores were available for analysis, the individual components contributing to the Apgar score could not be reliably extracted from the electronic medical records and therefore were not evaluated separately.

## 4. Materials and Methods

### 4.1. Study Design and Setting

We conducted a 12-year retrospective comparative cohort study including pregnant patients diagnosed with UUTIs in the Urology Clinic of “C.I. Parhon” University Hospital, Iași, Romania, between 1 January 2014 and 31 December 2025. This study was conducted and reported according to the RECORD (REporting of studies Conducted using Observational Routinely Collected Health Data) statement, an extension of the STROBE guidelines for studies using routinely collected health data. Patients who received meropenem during hospitalization were included in the meropenem group, while those treated with ceftriaxone during the same period constituted the ceftriaxone group. Patients who subsequently delivered at the tertiary maternity hospital “Cuza Vodă”, Iași, Romania, underwent follow-up until delivery and the early postpartum period.

Given the retrospective design, treatment allocation was not randomized. The decision to administer meropenem or ceftriaxone was made by the attending urologist or intensive care physician according to clinical judgment and disease severity. In clinical practice, meropenem was preferentially administered in patients presenting with more severe infections, particularly sepsis or septic shock, introducing potential confounding by indication.

The study was approved by the ethics committees of “C.I. Parhon” University Hospital (no. 1402/12.02.2025) and “Cuza Vodă” Maternity Hospital (no. 120/08.01.2026).

### 4.2. Patient Selection

Data were extracted from the electronic hospital records of “C.I. Parhon” Hospital using ICD-10 coding from the International Classification of Diseases, 10th edition [63], specifically code O09 (supervision of high-risk pregnancy). A total of 311 pregnant patients were initially identified.

Of these, 42 patients with gestational hydronephrosis and/or reno-ureteral lithiasis without confirmed UTIs and 9 patients with lower UTIs were excluded, leaving 260 patients with UUTI. Among these, 87 patients treated with amoxicillin–clavulanic acid or cefuroxime were excluded because these regimens were generally used in less severe infections and their inclusion would have introduced substantial heterogeneity into the comparator group. Ceftriaxone was selected as the comparator because it was more frequently administered in patients with severe UUTIs requiring hospitalization and therefore represented the most clinically relevant comparison group for evaluating meropenem use. One additional case was excluded because meropenem was administered in combination with vancomycin.

**Figure 1:**
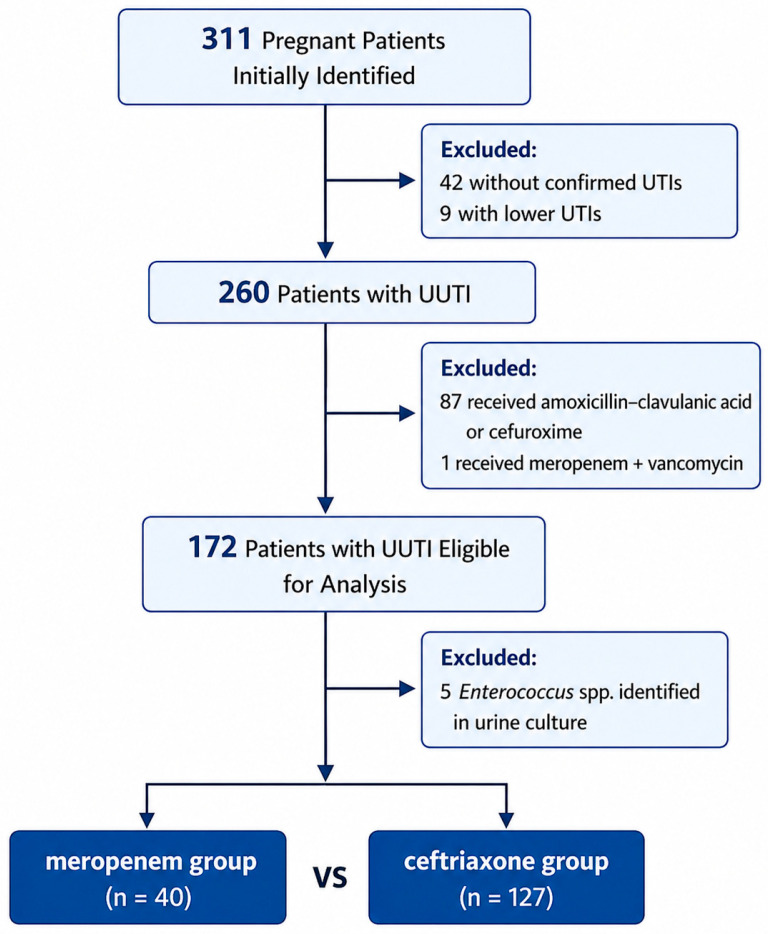
Patient selection. UTIs—urinary tract infections; UUTI—upper urinary tract infection.

A total of 172 patients with UUTI treated with either meropenem or ceftriaxone remained eligible for analysis. Five patients initially treated with ceftriaxone were excluded because urine culture identified *Enterococcus* spp., which are intrinsically resistant to cephalosporins. The final study population consisted of patients treated with meropenem (study group) and those treated with ceftriaxone (control group). The patient selection process is illustrated in Figure 1.

### 4.3. Antibiotic Therapy and Urological Procedures

In eight patients, initial treatment consisted of ceftriaxone (five patients) or amoxicillin–clavulanic acid (three patients), but therapy was subsequently changed to meropenem because of unfavorable clinical evolution. The decision to escalate antibiotic therapy was made by the attending physician based on persistent inflammatory syndrome, clinical deterioration, or the development of sepsis or septic shock, representing routine clinical practice in our institution for severe UUTIs during pregnancy. In four patients initially treated with ceftriaxone and two treated with amoxicillin–clavulanic acid, JJ stent insertion was performed. During or immediately after the procedure, these patients developed hemodynamic instability and septic shock, which led to the initiation of meropenem therapy. Two additional patients without urinary obstruction developed sepsis under initial antibiotic treatment and were therefore switched to meropenem. In all these cases, the change in antibiotic therapy occurred within the first 24 h after initiation of treatment, after administration of only one or two doses of the initial antibiotic.

In cases of obstructive pyelonephritis, antibiotic therapy was associated with ureteral catheterization and JJ stent insertion or percutaneous nephrostomy (one case). In patients with reflux pyelonephritis, JJ stents were replaced after initiation of antibiotic therapy.

### 4.4. Antibiotic Administration

In all patients, antibiotic therapy was initiated during hospitalization in the urology clinic, usually within the first hour after admission.

Empiric antibiotic therapy in patients with suspected sepsis or septic shock was primarily directed against Gram-negative uropathogens. Patients in the ceftriaxone group whose urine cultures subsequently identified *Enterococcus* spp. were switched to alternative antibiotic therapy and excluded from the final analysis because of the intrinsic resistance of enterococci to cephalosporins. In the meropenem group, all positive urine cultures identified Gram-negative pathogens.

Meropenem was administered intravenously as a slow infusion over 30 min at a dose of 1 g every 8 h. The formulations used were Meropenem (Antibiotice, Iași, Romania) or ARCHIFAR (Medochemie, Limassol, Cyprus).

Ceftriaxone was administered intravenously as a slow injection over a maximum of 5 min at a dose of 1–2 g every 12 h. The formulations used were Cefort (Antibiotice, Iași, Romania) or Seftrion (E.I.P.I.C.O.MED, Bucharest, Romania).

Both antibiotics were administered as monotherapy. After obtaining urine culture and antibiogram results, therapy was either continued or de-escalated to another injectable or oral antibiotic when appropriate and when favorable clinical evolution was observed. However, in patients with prolonged or severe clinical evolution, persistent inflammatory syndrome, sepsis, or septic shock, meropenem was continued despite susceptibility to alternative agents until clinical and inflammatory parameters improved.

### 4.5. Variables Analyzed

Comparative analyses between the two groups included maternal age, side of infection (right/left), place of origin (urban/rural), trimester of pregnancy at diagnosis, parity, and comorbidities (diabetes mellitus and anemia).

We also analyzed the clinical conditions leading to antibiotic therapy, including infected gestational hydronephrosis, infected hydronephrosis secondary to reno-ureteral lithiasis, reflux pyelonephritis, non-obstructive pyelonephritis, and the presence of sepsis or septic shock.

Clinical and laboratory variables included leukocyte count, C-reactive protein (CRP), serum creatinine, fever above 38 °C, transfer to the gynecology department, length of hospitalization, duration of antibiotic therapy, and the presence of MDR pathogens. MDR pathogens were defined according to the international consensus criteria proposed by Magiorakos et al. as acquired non-susceptibility to at least one agent in three or more antimicrobial categories [64]. In all patients, urine cultures were obtained at hospital admission before initiation of antibiotic therapy, according to the routine clinical protocol of our institution.

Clinical cure was defined as resolution of fever and lumbar pain, clearance of turbid urine, normalization of leukocyte count, and reduction in CRP levels. Microbiological cure was assessed based on readmission rates associated with recurrence of infection caused by the same pathogen.

### 4.6. Microbiology and Pregnancy Outcomes

The pathogens responsible for UUTI were analyzed comparatively between the two groups. Adverse reactions related to antibiotic therapy and possible discontinuation of treatment were also evaluated.

Patients from both groups were subsequently identified in the electronic records of “Cuza Vodă” Obstetrics and Gynecology Hospital, the largest maternity hospital in eastern Romania. For patients who delivered at this institution, pregnancy outcomes were analyzed, including birth weight, Apgar score, type of delivery (vaginal or cesarean), preterm birth, gestational age at delivery, and maternal or fetal mortality.

### 4.7. Diagnostic Criteria

UTI was defined by the presence of leukocyturia and nitrites on urinalysis and/or a positive urine culture. Patients with negative urine culture but abnormal urinalysis together with clinical and laboratory signs of infection were also included, although microbiological comparisons were performed only in patients with positive cultures. A urine culture was considered positive when bacterial growth exceeded 100,000 CFU/mL. A detailed comparative analysis of ESBL production and complete antimicrobial susceptibility profiles was beyond the primary scope of the present study, which focused mainly on the proportion of MDR pathogens and the clinical indications for meropenem therapy.

The diagnosis of UUTI was established based on the combination of clinical findings, laboratory investigations, and imaging studies. Clinical criteria included flank pain, fever, chills, cloudy urine, and lower urinary tract symptoms. Laboratory criteria included inflammatory syndrome and pathological urinalysis findings such as leukocyturia and/or positive nitrites. Positive urine culture was not mandatory for inclusion, as microbiological confirmation is not identified in all patients with UUTIs in routine clinical practice. Only patients with positive urine cultures were included in the microbiological analysis presented in Table 5. Sepsis and septic shock were defined according to the Sepsis-3 criteria [65]. Sepsis was defined as life-threatening organ dysfunction associated with infection, while septic shock was defined as sepsis associated with persistent hypotension requiring vasopressor support despite adequate fluid resuscitation. The qSOFA score was used as part of the clinical assessment of organ dysfunction. Reflux pyelonephritis was diagnosed when signs of UUTI occurred in patients carrying a JJ stent.

All patients were admitted as emergency cases and underwent urinary tract ultrasound at admission to identify hydronephrosis and its potential causes, such as reno-ureteral lithiasis, gestational hydronephrosis, or congenital urinary tract abnormalities. During hospitalization, all patients were evaluated by an obstetrician. In cases of suspected fetal distress, the patient was transferred to the obstetrics clinic of “Cuza Vodă” Hospital.

Pregnancy trimesters were defined as follows: first trimester (weeks 1–13), second trimester (weeks 14–27), and third trimester (weeks 28–40).

### 4.8. Statistical Analysis

Quantitative variables were expressed as mean ± standard deviation, while categorical variables were presented as percentages. Comparisons between normally distributed continuous variables were performed using Student’s *t* test, whereas non-parametric data were analyzed using the Mann–Whitney test. Categorical variables were compared using the chi-square test or Fisher’s exact test when appropriate. A *p* value < 0.05 was considered statistically significant. Statistical analyses were performed using SPSS version 24.0. Multivariate logistic regression analysis was performed to identify factors independently associated with meropenem administration. In the multivariate analysis, we included all major clinical indications for antibiotic therapy (infected gestational hydronephrosis, urolithiasis with hydronephrosis, reflux pyelonephritis, sepsis, and septic shock), as well as other variables that showed significant differences between the two groups in the univariate analysis (anemia, multiparity, and third trimester of pregnancy). Because of the relatively small size of the meropenem group, a maximum of five variables were included in each multivariate model to avoid overfitting and ensure model stability.

## 5. Conclusions

Pyelonephritis associated with urinary obstruction caused by gestational hydronephrosis or reno-ureteral lithiasis, particularly when complicated by sepsis or septic shock, represents the main indication for meropenem administration in pregnant patients with UUTIs. In our cohort, meropenem appeared to be associated with favorable maternal clinical outcomes and was well tolerated, with no reported adverse effects. However, its use did not eliminate the higher rate of transfer to obstetric care, most likely reflecting the greater severity of infection in these patients. Prospective studies including larger patient cohorts are required to further clarify the safety and effectiveness of meropenem therapy during pregnancy.

## Figures and Tables

**Table 1:** Patient characteristics in the two study groups.

Variable	Meropenem Group (40 Patients) *n* (%)/Mean ± SD	Ceftriaxone Group (127 Patients) *n* (%)/Mean ± SD	*p*
Age (years)	25.80 ± 6.86	25.66 ± 5.83	0.89
Location of infection			
Right	35 (87.5%)	95 (74.8%)	0.09
Left	5 (12.5%)	32 (25.2%)	
Trimester of pregnancy			
1st trimester	2 (5%)	12 (9.44%)	0.52
2nd trimester	15 (37.5%)	71 (55.9%)	0.042
3rd trimester	23 (57.5%)	44 (34.64%)	0.01
Place of origin			
Urban	21 (52.5%)	53 (41.73%)	0.25
Rural	19 (47.5%)	74 (58.26%)	
Parity			
Nulliparous	20 (50%)	93 (73.22%)	0.006
Multiparous	20 (50%)	34 (26.77%)	
Comorbidities			
Anemia	29 (72.5%)	70 (55.11%)	0.051
Diabetes mellitus	2 (5%)	1 (0.7%)	0.14

**Table 2:** Conditions for which antibiotic therapy was indicated.

Condition	Meropenem Group (*n* = 40)	Ceftriaxone Group (*n* = 127)	OR	95% CI for OR	RR	95% CI for RR	*p*-Value
Infected gestational hydronephrosis	25 (62.5%)	87 (68.50%)	0.77	0.37 to 1.61	0.91	0.70 to 1.19	0.44
Urolithiasis with hydronephrosis	7 (17.5%)	24 (18.90%)	0.91	0.36 to 2.30	0.93	0.43 to 1.99	0.82
Reflux pyelonephritis	5 (12.5%)	5 (3.94%)	3.49	0.95 to 12.73	3.17	0.97 to 10.41	0.062
Non-obstructive pyelonephritis	3 (7.5%)	7 (5.51%)	1.39	0.34 to 5.65	1.36	0.37 to 5.02	0.704
Sepsis Of which septic shock	36 (90.0%)	49 (38.58%)	14.33	4.80 to 42.74	2.33	1.83 to 2.97	<0.01
	12 (30.0%)	7 (5.51%)	7.35	2.65 to 20.35	5.44	2.30 to 12.89	<0.01

OR—odds ratio; RR—risk ratio; CI—confidence interval.

**Table 3:** Multivariate analysis of factors associated with the indication for meropenem therapy.

Variable	OR (95% CI)	*p*
Sepsis	10.54 (3.30–33.70)	<0.001
Septic shock	3.28 (1.01–10.62)	0.048

Abbreviations: OR—odds ratio; CI—confidence interval.

**Table 4:** Clinical and laboratory characteristics of the patients in the two groups.

Variable	Meropenem Group (*n* = 40)	Ceftriaxone Group (*n* = 127)	Effect Size	95% CI	*p*-Value
Leukocytes	18,396 ± 7038.2	15,247 ± 5152.2	Mean difference = 3149.00	735.71 to 5562.29	<0.01
CRP	177.4 ± 91.7	82.2 ± 73.8	Mean difference = 95.20	63.33 to 127.07	<0.01
Creatinine	0.935 ± 0.83	0.64 ± 0.38	Mean difference = 0.30	0.02 to 0.57	0.036
Length of hospitalization (days)	5.38 ± 1.9	4.38 ± 1.7	Mean difference = 1.00	0.33 to 1.67	<0.01
Duration of antibiotic therapy (days)	4.82 ± 2.2	4.11 ± 1.99	Mean difference = 0.71	−0.07 to 1.49	0.031
Fever > 38 °C	33 (82.5%)	56 (44.1%)	OR = 5.98	2.46 to 14.52	<0.01
Transfer to obstetrics care	8 (20.0%)	9 (7.08%)	OR = 3.28	1.17 to 9.18	0.032
MDR pathogens	9 (22.5%)	17 (13.38%)	OR = 1.88	0.76 to 4.62	0.16
Readmissions after treatment with positive urine culture	5 (12.5%)	11 (8.66%)	OR = 1.51	0.49 to 4.63	0.47
Associated urological surgical interventions with antibiotic treatment	37 (92.5%)	121 (95.27%)	OR = 0.61	0.15 to 2.57	0.99

CRP—C-reactive protein; MDR—multidrug resistant; OR—Odds Ratio; CI—confidence interval.

**Table 5:** Microorganisms responsible for UUTIs in the two groups.

Pathogen	Meropenem Group (32 Patients) *n*(%)	Ceftriaxone Group (86 Patients) *n* (%)	Odds Ratio (95% CI)	*p*-Value (Fisher)
*Escherichia coli*	23 (57.5%)	63 (49.6%)	1.37 (0.67–2.77)	0.468
*Klebsiella pneumoniae*	8 (20%)	9 (7.1%)	3.28 (1.2–8.9)	0.031
*Pseudomonas* spp.	0 (0%)	1 (0.8%)	0.0 (0.04–26.06)	1.0
*Proteus* spp.	0 (0%)	3 (2.4%)	0.0 (0.02–8.68)	1.0
*Serratia* spp.	0 (0%)	1 (0.8%)	0.0 (0.04–26.06)	1.0
*Staphylococcus aureus*	1 (2.5%)	4 (3.1%)	0.79 (0.16–6.85)	1.0
*Enterococcus* spp.	0 (0%)	5 (3.9%)	0.0 (0.01–5.08)	0.339

**Table 6:** Pregnancy outcomes in the two groups.

Variable	Meropenem Group (*n* = 33)	Ceftriaxone Group (*n* = 89)	Effect Size	95% CI	*p*-Value
Birth weight	3043.5 ± 524.8	3122.8 ± 469.4	Mean difference = −79.30	−288.03 to 129.43	0.414
Apgar score	7.73 ± 1.7	8.64 ± 1.20	Mean difference = −0.91	−1.56 to −0.26	0.007
Gestational age at birth	37.64 ± 2.13	38.06 ± 1.91	Mean difference = −0.42	−1.27 to 0.43	0.325
Vaginal delivery	17 (51.5%)	46 (51.7%)	OR = 0.99	0.45 to 2.21	1
Cesarean section	16 (48.5%)	43 (48.3%)	OR = 1.01	0.45 to 2.24	1
Preterm delivery < 38 weeks	10 (30.3%)	19 (21.3%)	OR = 1.60	0.65 to 3.94	0.428
Delivery ≥ 38 weeks	23 (69.7%)	70 (78.7%)	OR = 0.62	0.25 to 1.53	0.428
Stillbirth	2 (6.06%)	1 (1.1%)	OR = 5.68	0.50 to 64.82	0.17
NICU admission	23 (69.7%)	46 (51.7%)	OR = 2.15	0.92 to 5.03	0.10

## Data Availability

The original contributions presented in this study are included in the article/Supplementary Materials. Further inquiries can be directed to the corresponding author.

## Supplementary Materials

The following supporting information can be downloaded at: https://www.mdpi.com/article/10.3390/antibiotics15060610/s1. Table S1: Antibiotic Resistance Patterns of Bacterial Isolates Identified in the Study Population; The RECORD statement—checklist of items, extended from the STROBE statement, that should be reported in observational studies using routinely collected health data [66].
