# Supplementary material for: Efficacy and Safety of Meropenem in Pregnant Women with Upper Urinary Tract Infections: A Retrospective Cohort Study in Romania
Source: Antibiotics (Basel). 2026 Jun 16;15(6):610. doi: 10.3390/antibiotics15060610 (PMC13296016; doi:10.3390/antibiotics15060610)
Supplement: Supplementary file 1 [file antibiotics-15-00610-s001.zip › antibiotics-4321975-Table S1-.pdf]

Supplementary Table S1. Antibiotic Resistance Patterns of Bacterial Isolates Identified in the Study Population

Antibiotic	Meropenem group (32 patients) N (%)	Ceftriaxone group (86 patients) N(%)	OR (IC 95%)	p	Test
Ampicillin	23/32 (71.88%)	54/86 (62.79%)	1.51 (0.62-3.67)	0.357	Chi-square
Amoxicillin-clavulanic acid	9/32 (28.12%)	19/86 (22.09%)	1.38 (0.55-3.48)	0.494	Chi-square
Trimethoprim-sulfamethoxazole	12/32 (37.5%)	26/86 (30.23%)	1.38 (0.59-3.24)	0.453	Chi-square
Nitrofurantoin	3/32 (9.38%)	5/86 (5.81%)	1.68 (0.38-7.46)	0.681	Fisher exact
Piperacillin-tazobactam	1/32 (3.12%)	1/86 (1.16%)	2.74 (0.17-45.19)	0.471	Fisher exact
Imipenem	0/32 (0.0%)	0/86 (0.0%)	NA	NA	NA
Meropenem	0/32 (0.0%)	0/86 (0.0%)	NA	NA	NA
Cefuroxime	7/32 (21.88%)	13/86 (15.12%)	1.57 (0.56-4.38)	0.384	Chi-square
Ceftriaxone	6/32 (18.75%)	0/86 (0.0%)	42.43 (2.31-778.35)	<0.001	Fisher exact
Ceftazidime	6/32 (18.75%)	0/86 (0.0%)	42.43 (2.31-778.35)	<0.001	Fisher exact
Cefepime	5/32 (15.62%)	0/86 (0.0%)	34.6 (1.85-645.86)	0.001	Fisher exact
Ciprofloxacin	11/32 (34.38%)	18/86 (20.93%)	1.98 (0.81-4.85)	0.132	Chi-square
Levofloxacin	7/32 (21.88%)	11/86 (12.79%)	1.91 (0.67-5.46)	0.254	Fisher exact
Gentamicin	3/32 (9.38%)	5/86 (5.81%)	1.68 (0.38-7.46)	0.681	Fisher exact

Note: No resistance to imipenem or meropenem was detected among the bacterial isolates in either group; consequently, ORs, 95% CIs, and p-values were not calculable.
